# World Health Organization Global Estimates and Regional Comparisons of the Burden of Foodborne Disease in 2010

**DOI:** 10.1371/journal.pmed.1001923

**Published:** 2015-12-03

**Authors:** Arie H. Havelaar, Martyn D. Kirk, Paul R. Torgerson, Herman J. Gibb, Tine Hald, Robin J. Lake, Nicolas Praet, David C. Bellinger, Nilanthi R. de Silva, Neyla Gargouri, Niko Speybroeck, Amy Cawthorne, Colin Mathers, Claudia Stein, Frederick J. Angulo, Brecht Devleesschauwer, on behalf of World Health Organization Foodborne Disease Burden Epidemiology Reference Group

**Affiliations:** 1 National Institute for Public Health and the Environment, Bilthoven, The Netherlands; 2 University of Florida, Gainesville, Florida, United States of America; 3 Utrecht University, Utrecht, The Netherlands; 4 The Australian National University, Canberra, Australia; 5 University of Zurich, Zurich, Switzerland; 6 Gibb Epidemiology Consulting, Arlington, Virginia, United States of America; 7 Danish Technical University, Copenhagen, Denmark; 8 Institute of Environmental Science and Research, Christchurch, New Zealand; 9 Institute of Tropical Medicine, Antwerp, Belgium; 10 Boston Children's Hospital, Boston, Massachusetts, United States of America; 11 University of Kelaniya, Ragama, Sri Lanka; 12 Hikma Pharmaceuticals, Amman, Jordan; 13 Université catholique de Louvain, Brussels, Belgium; 14 World Health Organization, Geneva, Switzerland; 15 World Health Organization, Regional Office for Europe, Copenhagen, Denmark; 16 Centers for Disease Control and Prevention, Atlanta, Georgia, United States of America; 17 Ghent University, Merelbeke, Belgium; Mahidol-Oxford Tropical Medicine Research Unit, THAILAND

## Abstract

Illness and death from diseases caused by contaminated food are a constant threat to public health and a significant impediment to socio-economic development worldwide. To measure the global and regional burden of foodborne disease (FBD), the World Health Organization (WHO) established the Foodborne Disease Burden Epidemiology Reference Group (FERG), which here reports their first estimates of the incidence, mortality, and disease burden due to 31 foodborne hazards. We find that the global burden of FBD is comparable to those of the major infectious diseases, HIV/AIDS, malaria and tuberculosis. The most frequent causes of foodborne illness were diarrheal disease agents, particularly norovirus and *Campylobacter* spp. Diarrheal disease agents, especially non-typhoidal *Salmonella enterica*, were also responsible for the majority of deaths due to FBD. Other major causes of FBD deaths were *Salmonella* Typhi, *Taenia solium* and hepatitis A virus. The global burden of FBD caused by the 31 hazards in 2010 was 33 million Disability Adjusted Life Years (DALYs); children under five years old bore 40% of this burden. The 14 subregions, defined on the basis of child and adult mortality, had considerably different burdens of FBD, with the greatest falling on the subregions in Africa, followed by the subregions in South-East Asia and the Eastern Mediterranean D subregion. Some hazards, such as non-typhoidal *S*. *enterica*, were important causes of FBD in all regions of the world, whereas others, such as certain parasitic helminths, were highly localised. Thus, the burden of FBD is borne particularly by children under five years old–although they represent only 9% of the global population–and people living in low-income regions of the world. These estimates are conservative, i.e., underestimates rather than overestimates; further studies are needed to address the data gaps and limitations of the study. Nevertheless, all stakeholders can contribute to improvements in food safety throughout the food chain by incorporating these estimates into policy development at national and international levels.

## Abstract 2

An overview of foodborne diseases worldwide; part of the World Health Organization’s investigation into the incidence, mortality, and disease burden of foodborne hazards.

**Summary Points**

- Thirty-one foodborne hazards caused 600 (95% uncertainty interval [UI] 420–960) million foodborne illnesses and 420,000 (95% UI 310,000–600,000) deaths in 2010.
- The global burden of FBD caused by the 31 hazards studied was 33 (95% UI 25–46) million DALYs in 2010.
- The most frequent causes of foodborne illness were diarrheal disease agents; particularly norovirus and *Campylobacter* spp.
- Foodborne diarrheal disease agents, particularly non-typhoidal *Salmonella enterica*, caused 230,000 (95% UI 160,000–320,000) deaths
- Other major causes of FBD deaths were *Salmonella* Typhi, *Taenia solium*, hepatitis A virus and aflatoxin.
- 40% of the FBD burden was among children under 5 years old.
- The African (AFR), South-East Asian (SEAR) and Eastern Mediterranean (EMR) D subregions had the highest FBD burden.
- Diarrheal disease agents were the leading cause of FBD burden in most subregions, and non-typhoidal *Salmonella enterica* caused an important burden in all subregions, particularly in the subregions in Africa.
- Other main causes of diarrheal FBD burden were enteropathogenic *Escherichia coli*, enterotoxigenic *Escherichia coli* and *Vibrio cholerae* in low-income subregions, and *Campylobacter* spp. in high-income subregions.
- The burden of aflatoxin was high in the AFR D, Western Pacific (WPR) B and SEAR B subregions, whereas dioxins caused the highest burden in SEAR D, EMR D and European (EUR) A and C subregions.
- In the South-East Asian subregions, there was a considerable burden of *Salmonella* Typhi; the burden of *Opisthorchis* spp. was concentrated in the SEAR B region, where the seafoodborne trematodes *Paragonimus* spp. and *Clonorchis sinensis* were also important.
- In Central and South American (AMR B and AMR D) subregions, *T*. *solium* and *Toxoplasma gondii* contributed significantly to the FBD burden.
- These estimates should inform policy development at national and international levels to improve food safety throughout the food chain.

## Introduction

Illness and death from diseases caused by contaminated food are a constant threat to public health and a significant impediment to socio-economic development worldwide. Foodborne disease (FBD) outbreaks are common and often cause considerable morbidity and mortality. These diseases may be caused by infectious agents, such as the *Escherichia coli* O104:H4 outbreak attributed to contaminated fenugreek sprouts, which caused 386 cases of illness and 54 deaths in Germany in 2011 [1]. They can also be caused by chemical contamination, for example the melamine contamination of infant milk formula in China in 2008, which resulted in 294,000 cases of illnesses, 50,000 hospitalizations and at least 6 deaths [2]. Such incidents, although they capture widespread attention, constitute only a fraction of the global FBD burden.

Despite the evident importance of FBDs to public health, the full extent of chemical and biological contamination of food, and its cost to society, is still unknown. Contaminants in food were not even included as risk factors in previous studies of the global burden of all diseases [3]. Foodborne contaminants are numerous, however; they include viruses and bacteria, parasites, chemicals, toxins and allergens that cause a wide range of conditions. Many are pathogens that cause neglected tropical diseases, for which data is extremely limited. Indeed, epidemiological data on FBDs generally, particularly in the developing world, remain scarce. Outbreaks often go unrecognized, unreported or uninvestigated and may be visible only if they have a major public health or economic impact. Moreover, many foodborne hazards are also transmitted by other routes: through water, soil, or air; by direct contact between people, or by contact between people and animals. Determining the proportion of any illness that is foodborne can be difficult. Thus, estimating the global and regional burden of any FBD is complex.

Recognizing the need for global and regional estimates of FBDs to guide public health policy, in 2006 the World Health Organization (WHO) launched the ‘Initiative to Estimate the Global Burden of Foodborne Diseases’ [4]. The primary goal of this initiative is to enable policy makers and other stakeholders to set appropriate, evidence-based priorities in the area of food safety. To this end, the initiative aims to: improve the ability of countries to assess their burden of FBD; increase the number of countries who undertake these assessments; estimate the global burden, according to age, sex, and region, of FBDs caused by a defined list of biological and chemical agents; increase awareness of FBDs among WHO Member States and improve their commitment to implement food safety standards; and to encourage countries to use estimates of FBD to inform their policy making. To implement the initiative, in 2007 the WHO established the Foodborne Disease Burden Epidemiology Reference Group (FERG), an advisory group of external experts. The findings of this group are now being published in a collection of research papers in *PLOS Medicine* and *PLOS ONE* [5]. Here, we provide a summary of their findings, including estimates of the global and regional FBD burden obtained by the FERG; further details about the specific hazard groups, attribution methods, computational methods and country studies can be found in the accompanying collection of papers. A WHO Technical Report [6] also provides a comprehensive overview of methods, results, a description of the process and activities relating to capacity building in WHO member states.

### The FERG Task Forces

Several reviews have already described the background to the FERG and the approach it is taking [7–9]. In brief, the FERG consists of a Core (or Steering) Group to coordinate and oversee the scientific work, six 'task forces' advancing the work in specific areas, and external resource and technical advisers who are invited on an ad hoc basis to provide specific expertise. Three of the Task Forces are hazard-based (the Enteric Diseases Task Force, Parasitic Diseases Task Force, and Chemicals and Toxins Task Force), see Box 1. For each hazard that also has non-foodborne transmission pathway(s), a Source Attribution Task Force is specifically concerned with determining the proportion of the disease burden that is attributable to the consumption of contaminated food, see Box 2. The Country Studies Task Force fosters national FBD burden studies in selected countries (Albania, Japan, Thailand and Uganda) by piloting a specific protocol that was developed partly by adapting the National Burden of Disease Studies protocol published by the WHO [10]. The knowledge translation subgroup of this task force also provides tools to translate data on burden of disease into food safety policy. Finally, the Computational Task Force converts estimates from the hazard-based task forces into estimates of the global and regional burden of FBD, expressed in Disability Adjusted Life Years (DALYs), see Box 3.

**Box 1. Choice of Foodborne Hazards**

After reviewing the epidemiology of all the disease-causing agents potentially transmitted in food, the FERG hazard-based task forces identified 31 hazards (Table 1) to be included in the study, based on high incidence and/or mortality, and data availability. These included viruses, bacteria and protozoa causing predominantly acute diarrheal diseases (11 hazards); bacteria and protozoa causing invasive infectious diseases (seven hazards) and helminths (three cestodes, two nematodes and five trematodes including the broad group of ‘intestinal flukes’); and diseases induced by 3 chemical hazards. Analyses of the burden of arsenic, cadmium, lead and methylmercury in foods are ongoing and will be reported separately. Estimates of mortality for *Mycobacterium bovis* infections and morbidity and mortality for invasive non-typhoidal *Salmonella enterica* infections excluded illnesses attributed to HIV infection [11]. Incidence data for diseases caused by peanut allergens and four bacterial toxins (*Bacillus cereus*, *Clostridium botulinum*, *Clostridium perfringens* and *Staphylococcus aureus*) were not available for low-income countries and so were excluded from this global overview. Hazards occurring in particular regions, such as cyanide from cassava-based foods and foodborne trematodes, were included in the global overview.

**Table 1:** Hazards and outcomes included in estimates of the global burden of foodborne disease.

Hazards	Outcomes
**Diarrheal disease agents**	
Viruses	
Norovirus	diarrheal disease
Bacteria	
*Campylobacter* spp.	diarrheal disease, Guillain-Barré syndrome
Enteropathogenic *Escherichia coli*	diarrheal disease
Enterotoxigenic *E*. *coli*	diarrheal disease
Shiga toxin-producing *E*. *coli*	diarrheal disease, hemolytic uremic syndrome, end-stage renal disease
Non-typhoidal *Salmonella enterica*	diarrheal disease, invasive salmonellosis
*Shigella* spp.	diarrheal disease
*Vibrio cholerae*	diarrheal disease
Protozoa	
*Cryptosporidium* spp.	diarrheal disease
*Entamoeba histolytica*	diarrheal disease
*Giardia* spp.	diarrheal disease
**Invasive infectious disease agents**	
Viruses	
Hepatitis A virus	hepatitis
Bacteria	
*Brucella* spp.	acute brucellosis, chronic brucellosis, orchitis
*Listeria monocytogenes*	perinatal: sepsis, CNS^1^ infection, neurological sequelae
	acquired: sepsis, CNS infection, neurological sequelae
*Mycobacterium bovis*	tuberculosis
*Salmonella* Paratyphi A	paratyphoid fever, liver abcesses and cysts
*Salmonella* Typhi	typhoid fever, liver abcesses and cysts
Protozoa	
*Toxoplasma gondii*	congenital: intracranial calcification, hydrocephalus, CNS abnormalities, chorioretinitis early in life, chorioretinitis later in life
	acquired: chorioretinitis, acute illness, post-acute illness
**Helminths**	
Cestodes	
*Echinococcus granulosus*	pulmonary, hepatic, CNS cystic echinococcosis
*Echinococcus multilocularis*	abdominopelvic problems due to alveolar echinococcosis
*Taenia solium*	epilepsy
Nematodes	
*Ascaris* spp.	ascariasis, ascariasis-related mild abdominopelvic problems, ascariasis-related severe wasting
*Trichinella* spp.	acute clinical trichinellosis
Trematodes	
*Clonorchis sinensis*	abdominopelvic problems due to heavy clonorchiasis
*Fasciola* spp.	abdominopelvic problems due to heavy fascioliasis
Intestinal flukes^2^	abdominopelvic problems due to heavy intestinal fluke infection
*Opisthorchis* spp.	abdominopelvic problems due to heavy opistorchiasis
*Paragonimus* spp.	pulmonary problems due to heavy paragonimiasis, cerebral paragonimiasis
**Chemicals and toxins**	
Aflatoxin	hepatocellular carcinoma
Cassava cyanide	konzo
Dioxins	infertility, hypothyroidy due to prenatal and postnatal exposure

^1^ CNS: central nervous system

^2^ Includes selected species of the families *Echinostomatidae*, *Fasciolidae*, *Gymnophallidae*, *Heterophyidae*, *Nanophyetidae*, *Neodiplostomidae* and *Plagiorchiidae* (depending on data availability)

**Box 2. Attributing Illnesses to Food**

The Codex Alimentarius Commission (CAC) [12] defines food as: any substance, whether processed, semi-processed or raw, which is intended for human consumption, and includes drinks, chewing gum and any substance that has been used in the manufacture, preparation or treatment of food, but does not include cosmetics or tobacco or substances used only as drugs. The definition includes all bottled drinks. Food can be contaminated at many steps along the chain from farm/factory to plate so a precise definition of the point of attribution is crucial. Food can be contaminated at many steps along the chain from farm/factory to plate and in accordance with the CAC definition, FERG estimated the proportion of cases that are foodborne as those where food is contaminated just prior to consumption. Twelve of the 31 hazards were judged by the FERG hazard-based task forces to be 100% foodborne. To estimate the FBD burden for the remaining 19 hazards, a structured expert elicitation using Cooke’s Classical Method [13] was undertaken to distinguish the foodborne transmission pathway from environmental, human-to-human, and animal-to-human transmission pathways collectively. The study provided hazard-specific attribution estimates for each of the 14 subregions (see Table 2 for the countries in each subregion). Details on the FERG expert elicitation can be found elsewhere [14].

**Table 2:** World Health Organization (WHO) Member States by subregion.

Subregions^1^	WHO member states
AFR D	Algeria; Angola; Benin; Burkina Faso; Cameroon; Cape Verde; Chad; Comoros; Equatorial Guinea; Gabon; Gambia; Ghana; Guinea; Guinea-Bissau; Liberia; Madagascar; Mali; Mauritania; Mauritius; Niger; Nigeria; Sao Tome and Principe; Senegal; Seychelles; Sierra Leone; Togo.
AFR E	Botswana; Burundi; Central African Republic; Congo; Côte d'Ivoire; Democratic Republic of the Congo; Eritrea; Ethiopia; Kenya; Lesotho; Malawi; Mozambique; Namibia; Rwanda; South Africa; Swaziland; Uganda; United Republic of Tanzania; Zambia; Zimbabwe.
AMR A	Canada; Cuba; United States of America.
AMR B	Antigua and Barbuda; Argentina; Bahamas; Barbados; Belize; Brazil; Chile; Colombia; Costa Rica; Dominica; Dominican Republic; El Salvador; Grenada; Guyana; Honduras; Jamaica; Mexico; Panama; Paraguay; Saint Kitts and Nevis; Saint Lucia; Saint Vincent and the Grenadines; Suriname; Trinidad and Tobago; Uruguay; Venezuela (Bolivarian Republic of).
AMR D	Bolivia (Plurinational State of); Ecuador; Guatemala; Haiti; Nicaragua; Peru.
EMR B	Bahrain; Iran (Islamic Republic of); Jordan; Kuwait; Lebanon; Libyan Arab Jamahiriya; Oman; Qatar; Saudi Arabia; Syrian Arab Republic; Tunisia; United Arab Emirates.
EMR D	Afghanistan; Djibouti; Egypt; Iraq; Morocco; Pakistan; Somalia; South Sudan^2^; Sudan; Yemen.
EUR A	Andorra; Austria; Belgium; Croatia; Cyprus; Czech Republic; Denmark; Finland; France; Germany; Greece; Iceland; Ireland; Israel; Italy; Luxembourg; Malta; Monaco; Netherlands; Norway; Portugal; San Marino; Slovenia; Spain; Sweden; Switzerland; United Kingdom.
EUR B	Albania; Armenia; Azerbaijan; Bosnia and Herzegovina; Bulgaria; Georgia; Kyrgyzstan; Montenegro; Poland; Romania; Serbia; Slovakia; Tajikistan; The Former Yugoslav Republic of Macedonia; Turkey; Turkmenistan; Uzbekistan.
EUR C	Belarus; Estonia; Hungary; Kazakhstan; Latvia; Lithuania; Republic of Moldova; Russian Federation; Ukraine.
SEAR B	Indonesia; Sri Lanka; Thailand.
SEAR D	Bangladesh; Bhutan; Democratic People's Republic of Korea; India; Maldives; Myanmar; Nepal; Timor-Leste.
WPR A	Australia; Brunei Darussalam; Japan; New Zealand; Singapore.
WPR B	Cambodia; China; Cook Islands; Fiji; Kiribati; Lao People's Democratic Republic; Malaysia; Marshall Islands; Micronesia (Federated States of); Mongolia; Nauru; Niue; Palau; Papua New Guinea; Philippines; Republic of Korea; Samoa; Solomon Islands; Tonga; Tuvalu; Vanuatu; Viet Nam.

^1^ The subregions are defined on the basis of child and adult mortality as described by Ezzati et al. [15]. Stratum A: very low child and adult mortality, Stratum B: low child mortality and very low adult mortality, Stratum C: low child mortality and high adult mortality, Stratum D: high child and adult mortality, and Stratum E: high child mortality and very high adult mortality. The use of the term ‘subregion’ here and throughout the text does not identify an official grouping of WHO Member States, and the “subregions” are not related to the six official regions. AFR = African Region; AMR = Region of the Americas; EMR = Eastern Mediterranean Region; EUR = European Region; SEAR = South-East Asia Region; WPR = Western Pacific Region.

^2^ South Sudan was reassigned to the African Region in May 2013. As this study relates to time periods prior to this date, estimates for South Sudan were included in the Eastern Mediterranean Region.

**Box 3. Measures of Disease Burden**

The task forces estimated the incidence and duration of diseases caused by each hazard as well as their associated mortality in 2010 based on systematic reviews, complemented with other literature sources, surveillance data and expert inputs. For each hazard, the impact of all disease outcomes attributed to the primary exposure to a hazard is represented by a disease model. Two general approaches to estimate disease incidence were applied, guided by availability of data. The first approach aimed to identify all disease outcomes associated with a hazard and then establish probabilities for each outcome to occur. Any conditional dependencies between outcomes (based on the natural progression of the disease) were accounted for in this step. The second approach aimed to obtain data on the incidence of a specific outcome, and then to attribute a proportion of that incidence to a hazard. This hazard- and incidence-based approach is also used by the European Centre for Disease Prevention and Control to estimate the burden of a wide range of infectious diseases [16] and estimates the current and projected future burden of disease due to exposures occurring in 2010 or earlier. The chosen approach is well suited to illustrate the diversity of health impacts of FBD hazards, and highly relevant for identification of priorities for prevention, as reducing current exposure will also lead to prevention of future sequelae.

When incidence data were not available for all countries in a subregion, incidence rates were extrapolated from available data using a Bayesian log-normal random effects model, specifying the subregion as random effect. The predictive value of this model was explored and compared with other possible imputation models by McDonald et al. [17]. Detailed descriptions of methods to estimate incidence and mortality of all 31 hazards can be found in papers in the collection on enteric diseases [11], parasitic diseases [18], and chemicals and toxins [19].

The FERG studies used the DALY as a measure of population health to assess and compare the relative impact of different diseases and injuries on populations. The DALY measure combines the years of life lost (YLL) due to premature death and the years lived with disability (YLD) from a disease or condition, for varying degrees of severity, making time the common metric for death and disability [20]. One DALY is equivalent to one year of healthy life lost. As proposed by the Institute for Health Metrics and Evaluation in the Global Burden of Disease (GBD) 2010 study [21], and adopted by the WHO [22], we applied neither age-weighting nor time discounting. We used WHO life tables for YLL calculation. For YLD calculations, we used disability weights from the GBD 2010 study [23] with modifications as proposed by the WHO [22], and the GBD 2013 study [24]. DALY estimates were made at country level (194 countries) by 5-year age groups and gender for the base year 2010. Because of the level of uncertainty associated with estimates for each subpopulation, results are presented at subregional level (Table 2) for both sexes combined and for two age groups (<5 and ≥ 5 years of age). Both the population burden and the individual burden are presented. Statistical uncertainties in incidence and burden estimates were propagated by Monte Carlo simulation methods in the statistical programming environment R [25]. Full details of computational methods can be found in Devleesschauwer et al. [26]

### Global Disease Burden

Of the approximately 600 million cases of illness caused by the 31 foodborne hazards in 2010 (see Box 1 and Table 3), infectious agents that cause diarrheal diseases accounted for the vast majority (550 million), in particular norovirus (120 million cases) and *Campylobacter* spp. (96 million cases). Among other hazards, hepatitis A virus, the helminth *Ascaris* spp. and the typhoid bacterium *Salmonella* Typhi were frequent causes of foodborne illness, causing 14, 12 and 7.6 million cases, respectively.

**Table 3:** Median global number of foodborne illnesses, deaths, Years Lived with Disability (YLDs), Years of Life Lost (YLLs) and Disability Adjusted Life Years (DALYs), with 95% uncertainty intervals, 2010.

HAZARD	FOODBORNE ILLNESSES	FOODBORNE DEATHS	FOODBORNE YLDs	FOODBORNE YLLs	FOODBORNE DALYs
TOTAL	600,652,361 (417,646,804–962,834,044)	418,608 (305,128–598,419)	5,580,028 (4,780,374–8,195,314)	27,201,701 (19,655,451–38,922,210)	32,841,428 (24,809,085–46,274,735)
**Diarrheal disease agents**	**548,595,679 (369,976,912–888,528,014)**	**230,111 (160,039–322,359)**	**839,463 (644,924–1,123,907)**	**16,821,418 (11,700,916–23,579,652)**	**17,659,226 (12,458,675–24,516,338)**
Viruses	124,803,946 (70,311,254–251,352,877)	34,929 (15,916–79,620)	91,357 (51,047–174,130)	2,403,107 (1,102,397–5,387,672)	2,496,078 (1,175,658–5,511,092)
Norovirus	124,803,946 (70,311,254–251,352,877)	34,929 (15,916–79,620)	91,357 (51,047–174,130)	2,403,107 (1,102,397–5,387,672)	2,496,078 (1,175,658–5,511,092)
Bacteria	349,405,380 (223,127,469–590,002,559)	187,285 (131,742–254,037)	685,212 (521,848–921,335)	13,795,606 (9,688,221–18,893,580)	14,490,808 (10,303,551–19,681,271)
*Campylobacter* spp.	95,613,970 (51,731,379–177,239,714)	21,374 (14,604–32,584)	442,075 (322,192–587,072)	1,689,291 (1,141,055–2,652,483)	2,141,926 (1,535,985–3,137,980)
Enteropathogenic *E*. *coli*	23,797,284 (10,750,919–62,931,604)	37,077 (19,957–61,262)	22,977 (9,662–66,211)	2,908,551 (1,574,520–4,833,325)	2,938,407 (1,587,757–4,865,590)
Enterotoxigenic *E*. *coli*	86,502,735 (49,136,952–151,776,173)	26,170 (14,887–43,523)	70,567 (40,134–119,017)	2,011,635 (1,132,331–3,407,273)	2,084,229 (1,190,704–3,494,201)
Shiga toxin-producing *E*. *coli*	1,176,854 (754,108–2,523,007)	128 (55–374)	3,486 (1,741–6,996)	9,454 (4,140–27,208)	12,953 (5,951–33,664)
Non-typhoidal *S*. *enterica* ^1^	78,707,591 (31,843,647–211,154,682)	59,153 (36,341–89,045)	78,306 (35,961–185,179)	3,976,386 (2,410,953–6,180,921)	4,067,929 (2,486,092–6,271,290)
*Shigella* spp.	51,014,050 (20,405,214–118,927,631)	15,156 (6,839–30,072)	51,613 (21,184–114,267)	1,181,231 (519,372–2,445,834)	1,237,103 (554,204–2,520,126)
*Vibrio cholerae*	763,451 (310,910–1,567,682)	24,649 (10,304–50,042)	2,721 (1,019–6,020)	1,719,381 (718,642–3,487,195)	1,722,312 (720,029–3,491,997)
Protozoa	67,182,645 (35,794,977–120,556,797)	5,558 (2,593–11,958)	57,536 (30,526–102,608)	432,316 (195,372–960,910)	492,354 (239,400–1,034,790)
*Cryptosporidium* spp.	8,584,805 (3,897,252–18,531,196)	3,759 (1,520–9,115)	8,155 (3,598–17,355)	287,690 (114,012–711,990)	296,156 (119,456–724,660)
*Entamoeba histolytica*	28,023,571 (10,261,254–68,567,590)	1,470 (453–5,554)	20,851 (7,431–53,080)	115,740 (32,070–476,144)	138,863 (47,339–503,775)
*Giardia* spp.	28,236,123 (12,945,655–56,996,454)	0 (0–0)	26,270 (11,462–53,577)	0 (0–0)	26,270 (11,462–53,577)
**Invasive infectious disease agents**	**35,770,163 (18,604,754–70,045,873)**	**117,223 (54,789–243,482)**	**1,098,675 (729,530–1,796,607)**	**6,960,656 (3,128,316–14,882,637)**	**8,065,581 (3,983,949–16,557,714)**
Viruses	13,709,836 (3,630,847–38,524,946)	27,731 (7,169–77,320)	85,885 (22,118–250,641)	1,258,812 (325,409–3,509,844)	1,353,767 (383,684–3,672,726)
Hepatitis A virus	13,709,836 (3,630,847–38,524,946)	27,731 (7,169–77,320)	85,885 (22,118–250,641)	1,258,812 (325,409–3,509,844)	1,353,767 (383,684–3,672,726)
Bacteria	10,342,042 (3,506,116–27,627,480)	85,269 (37,573–196,544)	225,792 (108,092–604,162)	5,472,374 (2,283,968–12,803,285)	5,697,913 (2,394,245–13,384,811)
*Brucella* spp.	393,239 (143,815–9,099,394)	1,957 (661–45,545)	13,324 (4,095–315,952)	110,971 (37,470–2,583,081)	124,884 (43,153–2,910,416)
*Listeria monocytogenes*	14,169 (6,112–91,175)	3,175 (1,339–20,428)	2,255 (843–14,981)	116,109 (48,693–740,357)	118,340 (49,634–754,680)
*Mycobacterium bovis*	121,268 (99,852–150,239)	10,545 (7,894–14,472)	50,733 (38,441–68,052)	556,998 (417,711–761,851)	607,775 (458,364–826,115)
*Salmonella* Paratyphi A	1,741,120 (536,650–4,310,983)	12,069 (3,784–29,521)	26,987 (7,610–72,811)	829,136 (259,990–2,028,112)	855,730 (268,879–2,100,120)
*Salmonella* Typhi	7,570,087 (2,333,263–18,743,406)	52,472 (16,454–128,350)	117,334 (33,086–316,571)	3,604,940 (1,130,390–8,817,876)	3,720,565 (1,169,040–9,130,956)
Protozoa	10,280,089 (7,403,516–14,904,324)	684 (333–1,300)	763,326 (511,314–1,175,619)	62,899 (30,575–119,512)	829,071 (561,297–1,264,567)
*Toxoplasma gondii*	10,280,089 (7,403,516–14,904,324)	684 (333–1,300)	763,326 (511,314–1,175,619)	62,899 (30,575–119,512)	829,071 (561,297–1,264,567)
**Helminths**	**12,928,944 (8,957,617–24,008,256)**	**45,226 (34,143–59,035)**	**3,367,987 (2,840,638–4,358,741)**	**2,428,929 (1,869,610–3,173,545)**	**5,810,589 (4,864,518–7,367,619)**
Cestodes	430,864 (334,389–774,703)	36,500 (25,652–50,063)	1,220,578 (941,084–1,576,600)	1,932,154 (1,387,290–2,664,120)	3,158,826 (2,411,585–4,122,032)
*Echinococcus granulosus*	43,076 (25,881–371,177)	482 (150–3,974)	12,121 (5,515–99,213)	27,626 (8,577–227,715)	39,950 (16,996–322,953)
*Echinococcus multilocularis*	8,375 (656–17,005)	7,771 (243–15,896)	8,749 (856–22,576)	303,039 (8,102–622,954)	312,461 (9,083–640,716)
*Taenia solium*	370,710 (282,937–478,123)	28,114 (21,059–36,915)	1,192,236 (916,049–1,522,267)	1,586,288 (1,170,461–2,177,848)	2,788,426 (2,137,613–3,606,582)
Nematodes	12,285,286 (8,292,732–22,984,630)	1,012 (388–2,783)	518,451 (351,732–1,211,907)	80,021 (30,652–220,274)	605,738 (411,113–1,301,619)
*Ascaris* spp.	12,280,767 (8,287,414–22,980,491)	1,008 (384–2,781)	518,096 (351,418–1,211,691)	79,800 (30,426–220,154)	605,278 (410,668–1,301,114)
*Trichinella* spp.	4,472 (2,977–5,997)	4 (2–5)	342 (149–646)	210 (116–306)	550 (285–934)
Trematodes	218,569 (167,886–281,872)	7,533 (6,383–8,845)	1,616,785 (1,257,657–2,062,782)	403,884 (342,815–473,423)	2,024,592 (1,652,243–2,483,514)
*Clonorchis sinensis*	31,620 (21,515–45,059)	5,770 (4,728–6,988)	219,637 (149,514–312,718)	302,160 (247,586–366,036)	522,863 (431,520–635,232)
*Fasciola* spp.	10,635 (6,888–24,100)	0 (0–0)	90,041 (58,050–209,097)	0 (0–0)	90,041 (58,050–209,097)
Intestinal flukes^2^	18,924 (14,498–24,200)	0 (0–0)	155,165 (118,920–198,147)	0 (0–0)	155,165 (118,920–198,147)
*Opisthorchis* spp.	16,315 (11,273–22,860)	1,498 (1,230–1,813)	102,705 (70,849–143,938)	85,364 (70,123–103,317)	188,346 (151,906–235,431)
*Paragonimus* spp.	139,238 (95,610–195,078)	250 (160–371)	1,033,097 (730,118–1,423,031)	15,535 (9,971–23,035)	1,048,937 (743,700–1,438,588)
**Chemicals and toxins**	**217,632 (172,024–1,140,463)**	**19,712 (8,171–51,664)**	**247,920 (196,490–1,410,260)**	**650,157 (283,769–1,617,168)**	**908,356 (506,112–2,714,588)**
Aflatoxin	21,757 (8,967–56,776)	19,455 (7,954–51,324)	3,945 (1,551–10,667)	632,901 (265,578–1,606,493)	636,869 (267,142–1,617,081)
Cassava cyanide	1,066 (105–3,016)	227 (22–669)	2,521 (249–7,142)	15,694 (1,514–46,304)	18,203 (1,769–53,170)
Dioxins	193,447 (155,963–1,085,675)	0 (0–0)	240,056 (192,608–1,399,562)	0 (0–0)	240,056 (192,608–1,399,562)

^1^ Diarrheal and invasive disease

^2^ Includes selected species of the families *Echinostomatidae, Fasciolidae, Gymnophallidae, Heterophyidae, Nanophyetidae, Neodiplostomidae* and *Plagiorchiidae* (depending on data availability).

Foodborne diarrheal disease agents also caused 230,000 of the 420,000 deaths due to foodborne hazards (Table 3). Of these, non-typhoidal *S*. *enterica* accounted for 59,000, enteropathogenic *E*. *coli* (EPEC) for 37,000, norovirus for 35,000, and enterotoxigenic *E*. *coli* (ETEC) for 26,000 deaths. Of the 59,000 global deaths due to non-typhoidal *S*. *enterica*, 32,000 were in the two African subregions, and included 22,000 deaths due to invasive disease by this bacterium. The major non-diarrheal causes of foodborne deaths were due to *Salmonella* Typhi (52,000), the helminth *Taenia solium* (28,000) hepatitis A virus (28,000) and aflatoxin with 20,000 (95% UI 8,000–51,000) deaths.

**Fig 1:**
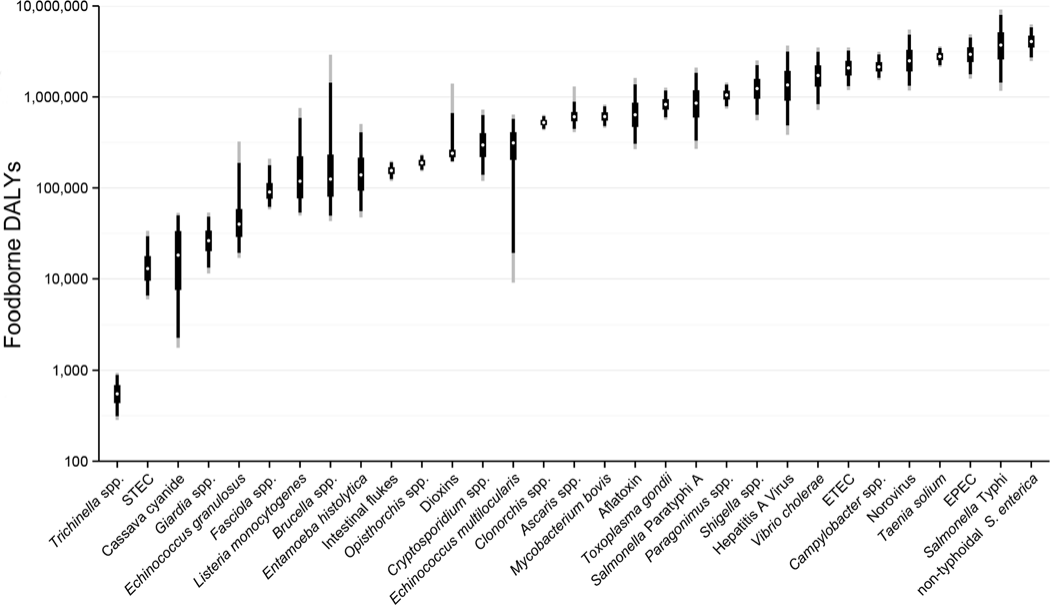
Ranking of foodborne hazards globally for 2010, expressed as Disability Adjusted Life Years. White dots indicate the median burden, black boxes the inter-quartile range (50% UI), black lines the 5 and 95 percentiles (90%UI) and grey lines the 2.5 and 97.5 percentiles (95% UI). Note the y-axis is on a logarithmic scale. Abbreviations: EPEC: Enteropathogenic *Escherichia coli*; ETEC: Enterotoxigenic *E*. *coli*; STEC: Shiga toxin-producing *E*. *coli*.

The global burden of FBD caused by the 31 hazards (including sequelae) in 2010 was 33 million DALYs (Table 3). Eighteen million DALYs, or 54%, of the total burden was attributed to diarrheal disease agents, particularly to non-typhoidal *S*. *enterica*, which was responsible for 4.0 million DALYs (Fig 1). Six diarrheal disease agents (norovirus, *Campylobacter* spp., EPEC, ETEC, *Vibrio cholerae* and *Shigella* spp.) each caused a foodborne burden of 1–3 million DALYs. Other foodborne hazards that contributed substantially to the global burden included *Salmonella* Typhi (3.7 million DALYs), *T*. *solium* (2.8 million DALYs), hepatitis A virus (1.4 million DALYs) and *Paragonimus* spp. (1.0 million DALYs). By contrast, the global burden of trichinellosis was estimated at only 550 DALYs. For full details of the numbers of cases of foodborne illness, deaths, DALYs, YLLs and YLDs for all 31 hazards in this study, see S1 Table, tabs 1–13. The Supporting Information also includes the data for total illnesses, deaths, DALYs, YLLs and YLDs by all exposure pathways for all hazards that were included in the source attribution expert elicitation (see S1 Table, tabs 14–18).

**Fig 2:**
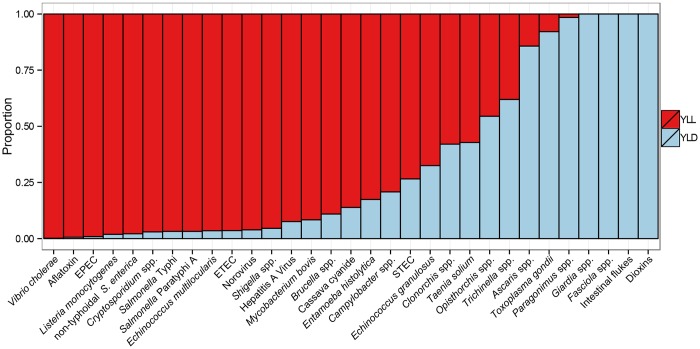
Relative contribution of years of life lost (YLL) due to premature mortality and years lived with disability (YLD) to the global burden of 31 hazards in food for 2010. For abbreviations, see Fig 1.

The relative contribution of mortality (measured as YLL) and morbidity (measured as YLD) to the total burden of disease varied widely between hazards (Fig 2). For 18 foodborne hazards, more than 75% of the total burden was due to premature mortality (red columns in Fig 2). These mainly include hazards leading to diseases with known high case-fatality ratios (non-typhoidal *S*. *enterica*, EPEC, ETEC, *Shigella* spp. and *V*. *cholerae*, *Listeria monocytogenes*, *Salmonella* Typhi and *Salmonella* Paratyphi, *Echinococcus multilocularis* and aflatoxin). At the other extreme, more than 75% of the total burden due to morbidity (blue columns in Fig 2) were accounted for by seven foodborne hazards, of which four (*Giardia* spp., *Fasciola* spp., intestinal flukes, and dioxins) were not assumed to cause fatal illnesses.

**Fig 3:**
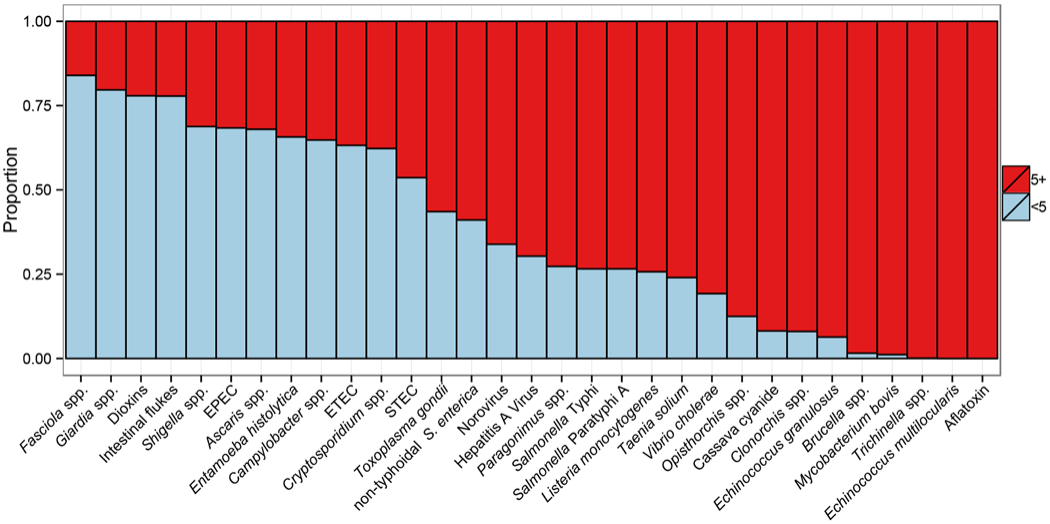
Age-distribution of Disability Adjusted Life Years for 31 hazards contributing to the global burden of foodborne disease for 2010. For abbreviations, see Fig 1.

The FERG studies show that children under five years old bear 40% of the foodborne disease burden (including, for some hazards, the life-long burden of sequelae). More than 75% of the burden of four hazards (*Fasciola* spp., *Giardia* spp., dioxins, and intestinal flukes) occurred among children under five (Fig 3). Prenatal infections accounted for 21% of the burden of *L*. *monocytogenes* [27] and for 32% of the burden of *Toxoplasma gondii* [18]. By contrast, more than 75% of the burden of 11 hazards occurred among people over five years old.

**Fig 4:**
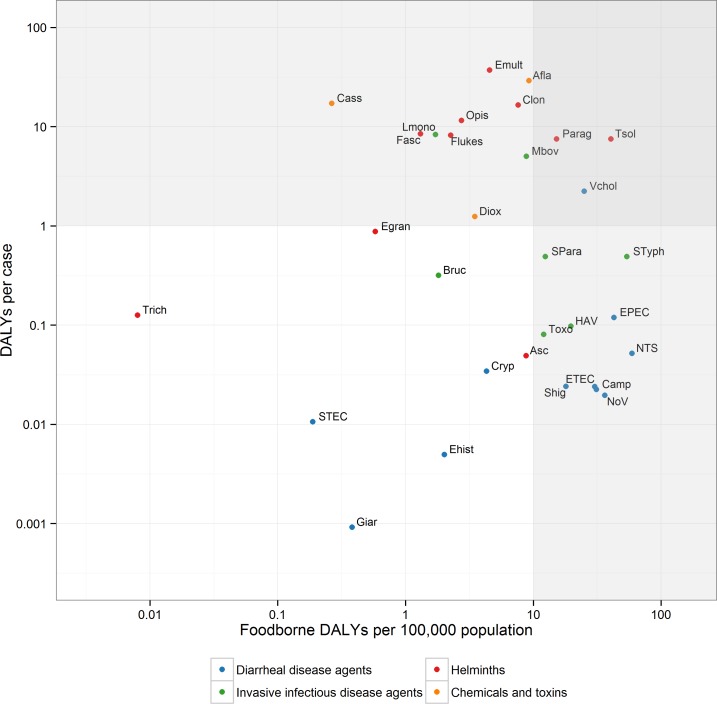
Scatterplot of the global burden of foodborne disease per 100,000 population and per case. The grey-shaded areas indicate arbitrary cut-offs between high (H) or low (L) population burden (> or ≤ 10 DALYs per 100,000 population) and high or low individual burden (> or ≤ 1 DALY per case). **Abbreviations:** NoV: Norovirus; Camp: *Campylobacter* spp.; EPEC: Enteropathogenic *Escherichia coli*; ETEC: Enterotoxigenic *E*. *coli*; STEC: Shiga toxin-producing *E*. *coli*; NTS: non-typhoidal *Salmonella enterica*; Shig: *Shigella* spp.; Vchol; *Vibrio cholerae* Ehist: *Entamoeba histolytica*; Cryp: *Cryptosporidium* spp.; Giar: *Giardia* spp.; HAV: Hepatitis A virus; Bruc: *Brucella* spp.; Lmono: *Listeria monocytogenes*; Mbov: *Mycobacterium bovis*; SPara: *Salmonella* Paratyphi A; STyph: *Salmonella* Typhi; Toxo: *Toxoplasma gondii*; Egran: *Echinococcus granulosus*; Emult: *E*. *multilocularis*; Tsol: *Taenia solium*; Asc: *Ascaris* spp.; Trich: *Trichinella* spp.; Clon: *Clonorchis sinensis*; Fasc: *Fasciola* spp.; Flukes: Intestinal flukes; Opis: *Opisthorchis* spp.; Parag: *Paragonimus* spp.; Diox: Dioxins; Afla: Aflatoxin.

Fig 4 presents a scatterplot of the burden at individual level (DALYs per case, a measure for disease severity) and the burden at population level (foodborne DALYs per 100,000 population, also accounting for disease incidence). On the basis of this plot, hazards were divided by two criteria with arbitrary cut-offs as indicated by grey-shaded areas in the figure. *V*. *cholerae*, *T*. *solium* and *Paragonimus* spp. were in the high/high category. All other diarrheal disease agents were in the high/low category, except STEC, *E*. *histolytica* and *Giardia* spp. (low/low). The L/L category further included *Trichinella* spp. The low/high category contained agents that are of relatively low global impact but have a high impact on affected individuals. These included different parasites, particularly *E*. *multilocularis*, the invasive bacteria *Brucella* spp., *L*. *monocytogenes* and *M*. *bovis*. In subregions where the burden is higher than the global average, these agents are of specific relevance to policy makers.

### Regional Differences

**Fig 5:**
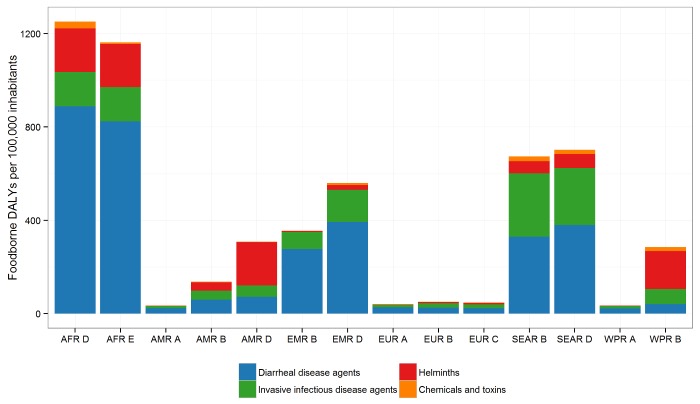
The global burden of foodborne disease (DALYS per 100,000 population) by hazard groups and by subregion for 2010. See Table 2 for the countries in each subregion.

The studies found considerable regional differences in the burden of FBD (Table 4 and Fig 5). The highest burden per 100,000 population was observed in the two African subregions: 1,300 DALYs per 100,000 population in AFR D and 1,200 DALYs per 100,000 population in AFR E. In the South-East Asian subregions, SEAR B and SEAR D, the burden was 690 and 710 DALYs per 100,000 population, respectively, and in the Eastern Mediterranean subregion, EMR D, 570 DALYs per 100,000 population. The lowest burden was observed in the North American subregion AMR A (35 DALYs per 100,000 population), followed by the three European subregions EUR A, EUR B and EUR C, and the Western Pacific subregion WPR A, which were all in the range of 40–50 DALYs per 100,000 population. Other subregions (AMR B and AMR D, EMR B and WPR B) had intermediate burdens, all in the range of 140–360 DALYs per 100,000 population (see Table 2 for a full list of the countries in each subregion).

**Table 4:** Median rates of foodborne Disability Adjusted Life Years (DALYs) per 100,000 population, by subregion, with 95% uncertainty intervals, 2010.

HAZARD	AFR D	AFR E	AMR A	AMR B	AMR D	EMR B	EMR D	EUR A	EUR B	EUR C	SEAR B	SEAR D	WPR A	WPR B
TOTAL	1,276 (459–2,263)	1,179 (726–1,764)	35 (23–49)	140 (97–274)	315 (243–575)	362 (205–582)	571 (325–954)	41 (29–64)	52 (33–136)	49 (33–77)	685 (360–1,291)	711 (343–1,335)	36 (23–170)	293 (219–406)
**Diarrheal disease agents**	**889 (196–1,731)**	**824 (447–1,326)**	**23 (13–33)**	**60 (36–94)**	**72 (40–117)**	**277 (153–460)**	**393 (217–644)**	**28 (17–39)**	**25 (14–37)**	**24 (13–35)**	**330 (154–576)**	**380 (159–717)**	**23 (14–32)**	**41 (21–65)**
Viruses	75 (6–222)	76 (0–225)	3 (0.6–8)	12 (0–38)	13 (0–43)	28 (0.5–79)	33 (0.4–90)	4 (0–11)	3 (0.08–9)	3 (0.2–9)	55 (0–224)	69 (0.8–263)	3 (0.09–7)	4 (0–19)
Norovirus	75 (6–222)	76 (0–225)	3 (0.6–8)	12 (0–38)	13 (0–43)	28 (0.5–79)	33 (0.4–90)	4 (0–11)	3 (0.08–9)	3 (0.2–9)	55 (0–224)	69 (0.8–263)	3 (0.09–7)	4 (0–19)
Bacteria	787 (186–1,482)	712 (393–1,160)	19 (10–28)	45 (26–68)	54 (28–87)	237 (124–403)	347 (190–576)	24 (14–32)	21 (11–32)	20 (10–29)	247 (107–429)	285 (119–506)	19 (11–27)	34 (17–54)
*Campylobacter* spp.	71 (35–119)	70 (33–117)	9 (5–14)	15 (8–23)	15 (8–26)	75 (40–109)	97 (54–143)	10 (6–14)	8 (4–13)	8 (4–12)	37 (14–87)	33 (2–84)	9 (5–14)	10 (4–17)
Enteropathogenic *E*. *coli*	136 (11–329)	138 (6–327)	0.006 (0.001–0.02)	7 (0.4–21)	9 (0.9–26)	46 (9–114)	60 (8–151)	0.006 (0.001–0.02)	0.004 (0–0.01)	0.004 (0–0.01)	64 (3–144)	65 (1–162)	0.006 (0.001–0.02)	5 (0.3–13)
Enterotoxigenic *E*. *coli*	107 (26–245)	105 (17–240)	0.003 (0–0.009)	7 (1–19)	9 (2–25)	29 (6–78)	37 (5–102)	0.004 (0–0.01)	0.004 (0–0.01)	0.004 (0–0.01)	42 (3–106)	42 (2–111)	0.003 (0–0.01)	4 (0.3–11)
Shiga toxin-producing *E*. *coli*	0.009 (0.002–0.03)	0.08 (0.02–0.2)	0.2 (0.05–0.3)	0.4 (0.09–1)	0.5 (0.2–1)	0.2 (0.09–0.4)	0.2 (0.1–0.5)	0.6 (0.2–1)	0.07 (0.02–0.2)	0.1 (0.03–0.4)	0.2 (0.02–1)	0.2 (0.007–1)	0.4 (0.1–1)	0.01 (0.002–0.04)
Non-typhoidal *S*. *enterica* ^1^	338 (94–612)	193 (44–336)	9 (4–16)	11 (2–20)	14 (4–26)	50 (17–82)	67 (26–112)	12 (7–18)	12 (6–21)	11 (5–19)	59 (22–154)	58 (0–162)	9 (5–14)	9 (4–16)
*Shigella* spp.	37 (0–156)	37 (0–148)	0.2 (0–1)	2 (0–8)	2 (0–9)	27 (0–109)	37 (0–145)	0.09 (0–0.9)	0.1 (0–1)	0.2 (0–1)	25 (0.6–84)	25 (0.7–90)	0.2 (0–1)	4 (0.1–11)
*Vibrio cholerae*	70 (2–197)	143 (4–383)	0 (0–0)	0 (0–0)	0 (0–0)	0.2 (0.009–0.5)	28 (0.9–96)	0 (0–0)	0 (0–0)	0 (0–0)	2 (0.2–3)	36 (0.2–133)	0 (0–0)	0.1 (0.005–0.4)
Protozoa	20 (0–74)	21 (5–66)	0.2 (0.01–1)	2 (0.4–6)	3 (0.5–8)	6 (0.9–27)	7 (1–33)	0.2 (0–0.8)	0.2 (0–0.9)	0.2 (0–0.9)	10 (2–35)	10 (2–39)	0.2 (0–0.9)	1 (0.2–4)
*Cryptosporidium* spp.	12 (0–44)	12 (0–45)	0.2 (0.01–0.9)	0.7 (0.08–3)	1 (0.1–5)	3 (0–21)	3 (0–24)	0.1 (0–0.8)	0.1 (0–0.8)	0.1 (0–0.8)	6 (0–28)	6 (0–32)	0.1 (0–0.8)	0.3 (0–3)
*Entamoeba histolytica*	5 (0–41)	5 (0–41)	0 (0–0)	0.4 (0–2)	0.4 (0–3)	2 (0–12)	2 (0–17)	0 (0–0)	0 (0–0)	0 (0–0)	2 (0–17)	2 (0–18)	0 (0–0)	0.3 (0–1)
*Giardia* spp.	0.7 (0–3)	0.7 (0–3)	0.03 (0–0.1)	0.4 (0–2)	0.5 (0.01–3)	0.4 (0–2)	0.6 (0.005–2)	0.03 (0–0.1)	0.03 (0–0.1)	0.03 (0–0.1)	0.1 (0–0.9)	0.1 (0–1)	0.03 (0–0.1)	0.3 (0–1)
**Invasive infectious disease agents**	**146 (46–342)**	**147 (55–343)**	**10 (6–14)**	**38 (16–76)**	**49 (19–144)**	**73 (32–148)**	**137 (38–334)**	**10 (7–15)**	**19 (9–61)**	**16 (10–29)**	**272 (71–721)**	**244 (38–623)**	**10 (5–132)**	**65 (19–145)**
Viruses	27 (4–77)	18 (3–55)	0.5 (0.07–2)	1 (0.1–4)	2 (0.2–7)	2 (0.2–5)	32 (2–102)	0.8 (0.03–2)	1 (0.2–3)	1 (0.3–4)	5 (0.6–15)	58 (6–182)	1 (0.07–3)	5 (0.3–17)
Hepatitis A virus	27 (4–77)	18 (3–55)	0.5 (0.07–2)	1 (0.1–4)	2 (0.2–7)	2 (0.2–5)	32 (2–102)	0.8 (0.03–2)	1 (0.2–3)	1 (0.3–4)	5 (0.6–15)	58 (6–182)	1 (0.07–3)	5 (0.3–17)
Bacteria	93 (31–259)	104 (40–277)	4 (2–7)	16 (4–47)	19 (5–65)	50 (16–121)	82 (22–241)	3 (3–5)	8 (3–39)	5 (3–10)	251 (59–696)	165 (27–490)	3 (1–126)	50 (12–124)
*Brucella* spp.	2 (0.2–53)	0.3 (0.007–18)	0.07 (0.02–0.6)	1 (0.3–16)	2 (0.2–38)	23 (3–83)	4 (0.6–68)	0.3 (0.07–1)	4 (0.7–35)	0.8 (0.07–6)	0.8 (0.004–112)	0.7 (0.003–92)	0.6 (0.02–125)	0.6 (0.09–9)
*Listeria monocytogenes*	1 (0–21)	1 (0–21)	3 (2–5)	2 (0.2–17)	1 (0–21)	1 (0–21)	1 (0–21)	3 (2–4)	0.3 (0.2–0.8)	0.6 (0.3–2)	1 (0–21)	1 (0–21)	1 (0.7–2)	1 (1–4)
*Mycobacterium bovis*	25 (15–39)	34 (21–48)	0.03 (0.01–0.06)	0.4 (0.2–0.8)	2 (0.8–4)	1 (0.5–3)	13 (6–25)	0.08 (0.06–0.1)	0.6 (0.5–1)	3 (2–5)	11 (4–27)	14 (6–27)	0.1 (0.08–0.2)	3 (1–5)
*Salmonella* Paratyphi A	11 (0–39)	12 (0–43)	0.1 (0–0.4)	2 (0–6)	2 (0.006–7)	3 (0–12)	10 (0–36)	0.02 (0–0.1)	0.3 (0–2)	0.01 (0–0.06)	42 (7–120)	26 (0.6–80)	0.1 (0–0.5)	8 (1–22)
*Salmonella* Typhi	47 (0–169)	52 (0–187)	0.4 (0–2)	7 (0–27)	8 (0.03–29)	14 (0–51)	45 (0–158)	0.09 (0–0.6)	2 (0–9)	0.04 (0–0.3)	184 (32–522)	113 (3–347)	0.6 (0–2)	36 (6–95)
Protozoa	21 (8–41)	20 (9–37)	5 (2–8)	20 (9–33)	27 (10–84)	20 (10–35)	18 (9–31)	6 (3–9)	10 (5–23)	10 (5–18)	13 (6–22)	9 (2–19)	5 (3–8)	9 (4–14)
*Toxoplasma gondii*	21 (8–41)	20 (9–37)	5 (2–8)	20 (9–33)	27 (10–84)	20 (10–35)	18 (9–31)	6 (3–9)	10 (5–23)	10 (5–18)	13 (6–22)	9 (2–19)	5 (3–8)	9 (4–14)
**Helminths**	**186 (125–308)**	**184 (141–240)**	**1 (0.9–4)**	**36 (27–134)**	**185 (149–229)**	**5 (2–15)**	**21 (12–40)**	**0.4 (0.2–1)**	**6 (3–27)**	**6 (4–15)**	**52 (42–64)**	**60 (45–80)**	**2 (1–3)**	**162 (131–202)**
Cestodes	172 (112–289)	178 (136–235)	0.4 (0.3–0.6)	25 (19–34)	71 (53–95)	1 (0.2–10)	0.7 (0.1–19)	0.2 (0.05–0.5)	4 (2–25)	4 (2–12)	3 (2–5)	46 (34–61)	0.03 (0.007–0.8)	45 (25–65)
*Echinococcus granulosus*	0.4 (0.06–21)	0.8 (0.2–16)	0.01 (0.002–0.03)	0.3 (0.02–5)	2 (0.4–8)	0.9 (0.2–10)	0.6 (0.1–19)	0.1 (0.02–0.4)	2 (0.5–6)	0.5 (0.09–1)	0.001 (0–0.1)	0.8 (0.2–3)	0.02 (0.001–0.8)	0.3 (0.08–0.9)
*Echinococcus multilocularis*	0 (0–0)	0 (0–0)	0 (0–0)	0 (0–0)	0 (0–0)	0.03 (0.005–0.06)	0.005 (0–0.05)	0.03 (0.008–0.06)	2 (0.5–21)	2 (0.5–11)	0 (0–0)	0.007 (0–0.04)	0.008 (0.001–0.02)	18 (0–37)
*Taenia solium*	170 (110–283)	176 (134–229)	0.4 (0.3–0.6)	25 (19–32)	69 (51–91)	0 (0–0)	0 (0–0)	0 (0–0)	0 (0–0)	0.9 (0.6–2)	3 (2–5)	45 (33–60)	0 (0–0)	27 (20–35)
Nematodes	13 (2–28)	5 (1–11)	0.6 (0.3–0.9)	11 (3–106)	12 (3–24)	3 (1–7)	13 (4–20)	0.04 (0.02–0.07)	1 (0.3–2)	1 (0.4–2)	8 (2–15)	13 (4–26)	0.004 (0.001–0.007)	11 (3–22)
*Ascaris* spp.	13 (2–28)	5 (1–11)	0.6 (0.3–0.9)	11 (3–106)	12 (3–24)	3 (1–7)	13 (4–20)	0 (0–0)	1 (0.3–2)	1 (0.3–2)	8 (2–15)	13 (4–26)	0 (0–0)	11 (3–22)
*Trichinella* spp.	0.001 (0–0.002)	0.001 (0–0.002)	0.009 (0.005–0.01)	0.009 (0.005–0.01)	0.009 (0.005–0.01)	0 (0–0)	0 (0–0)	0.04 (0.02–0.07)	0.04 (0.02–0.07)	0.04 (0.02–0.07)	0 (0–0.001)	0 (0–0.001)	0.004 (0.001–0.007)	0.004 (0.001–0.007)
Trematodes	0.06 (0.02–0.2)	0.02 (0.008–0.07)	0.2 (0.04–3)	0.1 (0.04–0.5)	101 (74–135)	0.3 (0.2–0.5)	7 (4–10)	0.2 (0.05–0.6)	0.2 (0.05–0.6)	1 (0.8–1)	40 (32–50)	0.7 (0.2–2)	2 (1–2)	106 (85–131)
*Clonorchis sinensis*	0 (0–0)	0 (0–0)	0 (0–0)	0 (0–0)	0 (0–0)	0 (0–0)	0 (0–0)	0 (0–0)	0 (0–0)	0.04 (0.03–0.04)	0.01 (0.003–0.04)	0.04 (0.01–0.2)	0.05 (0.01–0.2)	31 (26–38)
*Fasciola* spp.	0.02 (0.008–0.07)	0.01 (0.005–0.04)	0.04 (0.001–2)	0.04 (0.02–0.1)	46 (27–75)	0.2 (0.1–0.3)	7 (4–10)	0.07 (0.02–0.2)	0.06 (0.02–0.2)	0.04 (0.01–0.1)	0.02 (0.008–0.05)	0.05 (0.02–0.1)	0.07 (0.01–0.4)	0.9 (0.1–8)
Intestinal flukes^2^	0.01 (0.005–0.04)	0 (0–0)	0.1 (0.04–0.5)	0.06 (0.02–0.2)	0 (0–0)	0.06 (0.02–0.2)	0.08 (0.03–0.2)	0.03 (0.009–0.09)	0.05 (0.02–0.2)	0.09 (0.03–0.2)	0.2 (0.1–0.5)	0.1 (0.03–0.4)	1 (0.9–2)	9 (7–11)
*Opisthorchis* spp.	0 (0–0)	0 (0–0)	0 (0–0)	0 (0–0)	0 (0–0)	0 (0–0)	0 (0–0)	0.07 (0.02–0.3)	0.05 (0.01–0.3)	0.9 (0.6–1)	40 (32–50)	0.4 (0.1–1)	0 (0–0)	3 (2–4)
*Paragonimus* spp.	0.03 (0.008–0.08)	0.008 (0.002–0.02)	0.04 (0.004–0.6)	0.04 (0.01–0.1)	53 (38–73)	0 (0–0)	0.02 (0.008–0.07)	0 (0–0)	0 (0–0)	0.03 (0.01–0.1)	0.05 (0.008–0.5)	0.06 (0.02–0.2)	0.05 (0.02–0.2)	60 (43–83)
**Chemicals and toxins**	**30 (8–85)**	**7 (3–21)**	**0.4 (0.2–3)**	**3 (0.7–16)**	**2 (0.09–159)**	**0.8 (0.3–14)**	**9 (4–66)**	**2 (1–22)**	**0.9 (0.4–25)**	**2 (2–9)**	**20 (4–75)**	**18 (13–52)**	**0.3 (0.06–13)**	**18 (3–71)**
Aflatoxin	28 (7–78)	3 (1–8)	0.04 (0.006–0.2)	3 (0.6–9)	2 (0.07–137)	0.7 (0.2–3)	5 (1–17)	0.3 (0.1–0.7)	0.6 (0.3–1)	0.5 (0.2–2)	18 (3–52)	4 (0.6–15)	0.2 (0.04–0.8)	17 (3–69)
Cassava cyanide	1 (0.1–3)	3 (0.3–9)	0 (0–0)	0 (0–0)	0 (0–0)	0 (0–0)	0 (0–0)	0 (0–0)	0 (0–0)	0 (0–0)	0 (0–0)	0 (0–0)	0 (0–0)	0 (0–0)
Dioxins	0.2 (0.05–6)	0.2 (0.09–9)	0.3 (0.1–3)	0.1 (0.03–11)	0.2 (0.01–23)	0.09 (0.004–11)	3 (2–56)	2 (1–22)	0.3 (0.09–24)	2 (1–8)	0.2 (0.005–45)	14 (12–40)	0.1 (0.02–12)	0.06 (0.006–5)

^1^ Diarrheal and invasive disease.

^2^ Includes selected species of the families *Echinostomatidae, Fasciolidae, Gymnophallidae, Heterophyidae, Nanophyetidae, Neodiplostomidae* and *Plagiorchiidae* (depending on data availability).

The contribution of individual hazards to the burden of FBD differed markedly between subregions (Fig 5). In both African subregions, nearly 70% of the burden was due to diarrheal disease agents, particularly to non-typhoidal *S*. *enterica* (including invasive salmonellosis), EPEC, and ETEC; additionally, *V*. *cholerae* caused an important burden of diarrheal disease in the AFR E subregion, and *T*. *solium* caused a high burden in both African subregions (see Table 4 for the detailed data for all hazards and all subregions). In the SEAR D and SEAR B subregions, diarrheal disease agents contributed approximately 50% of the total disease burden, mainly caused by a range of hazards including EPEC, norovirus, non-typhoidal *S*. *enterica*, ETEC and *Campylobacter* spp. In both of these subregions, there was also a considerable burden of *Salmonella* Typhi (180 DALYs per 100,000 population in SEAR B and 110 DALYs per 100,000 population in SEAR D). The burden of disease due to the fluke *Opisthorchis* spp. was almost exclusively concentrated in SEAR B (40 DALYs per 100,000 population). In EMR D, diarrheal disease agents were responsible for approximately 70% of the total burden of FBD, with *Campylobacter* spp. the leading cause in the region, followed by EPEC, non-typhoidal *S*. *enterica*, *Shigella* spp. and ETEC. Other important hazards in this region were *Salmonella* Typhi, aflatoxin and hepatitis A virus.

In the WPR B subregion, diarrheal disease agents accounted for approximately 14% of the FBD burden, with *Campylobacter* spp. the leading cause. In this region, the seafoodborne trematodes *Paragonimus* spp. and *Clonorchis sinensis* were important contributors to the FBD burden. In the AMR B and AMR D subregions, the contribution of diarrheal disease agents to the total burden was smaller than in other subregions (approximately 40% and 20%, respectively), with *Campylobacter* spp., norovirus and non-typhoidal *S*. *enterica* causing most burden. In the AMR B region, important causes of FBD burden were *T*. *solium* (25 DALYs per 100,000 population) and *T*. *gondii* (20 DALYs per 100,000 population). In the AMR D region, the burden of *T*. *solium* was particularly high at 69 DALYs per 100,000 population; the trematodes *Paragonimus* spp. and *Fasciola spp*. contributing 53 and 46 DALYs per 100,000 population, respectively to the overall disease burden.

The burden due to chemical hazards was also highly localized. Aflatoxin caused the highest burden in AFR D, WPR B and SEAR B, whereas dioxins caused the highest burden in SEAR D, EMR D and EUR A and C. The burden of cassava cyanide was limited to the AFR regions, and was similar to that of aflatoxin in AFR D.

In the three European subregions, diarrheal disease agents contributed to 49–68% of the total burden of FBD, with non-typhoidal *S*. *enterica* and *Campylobacter* spp. being the most important hazards. Other important hazards included *T*. *gondii* in all European subregions, *Brucella* spp. in the EUR B and *Mycobacterium bovis* in the EUR C subregions. In the WPR A region, 65% of the burden was caused by diarrheal disease agents, with *T*. *gondii* and hepatitis A virus also contributing. Finally, in the AMR A region, diarrheal disease agents contributed approximately 67% of the total burden, with non-typhoidal *S*. *enterica* and *Campylobacter* spp. the most important hazards; *T*. *gondii* and *L*. *monocytogenes* were also relatively important.

### Previous Estimates

Several high-income countries have published national estimates of FBD. Estimates of food-related illnesses and deaths in the USA were reported in the late 1990s [28] and updated to cover the period 2000–2008 [29,30]. Similar studies are available from the UK [31], Australia [32], France [33] and Canada [34]. Some countries have extended this work to estimate DALYs, including New Zealand [35], Greece [36], the Netherlands [37], and the USA [38]. While the range of hazards covered in these previous studies differed from those of the FERG studies, the focus was on enteric diseases and a limited number of invasive and parasitic diseases. The FERG data, by contrast, cover numerous countries across the globe and provide a more complete picture of FBD.

Comparisons of our estimate of the burden of FBD with other estimates, such as those of the Institute for Health Metrics and Evaluation's GBD 2010 study [21], must be made with care because of differences in the methodology and data used. For example, the GBD 2010 study used prevalence-based DALYs, whereas our study used incidence-based DALYs. As a consequence, the impact of sequelae such as Guillain-Barré syndrome (due to *Campylobacter* spp.), hemolytic uremic syndrome (due to Shiga toxin-producing *E*. *coli*) and invasive disease (due to non-typhoidal *S*. *enterica*) were attributed to the diarrheal disease agents in our estimates whereas in the GBD 2010 study they were recorded in different disease categories. Furthermore, the GBD 2010 study used a different life table than FERG and more extensive mathematical modeling to account for data gaps, which smoothed the data considerably, resulting in narrower uncertainty intervals than in our study. The GBD 2010 and FERG studies used the same set of disability weights, but the FERG included some updates as recommended by WHO. Neither study applied time-discounting or age-weighting in their baseline estimates.

The GBD 2010 study, which looked at all sources of disease, found that the key hazards and risk factors for disease burden were dietary risk factors (254 million DALYs), unimproved water and sanitation (211 million DALYs), HIV/AIDS (82 million DALYs), malaria (82 million DALYs), air pollution (76 million DALYs) and tuberculosis (49 million DALYs). Recently published findings from WHO [39] for 2012 were: HIV/AIDS (92 million DALYs); malaria (55 million DALYs) and tuberculosis (44 million DALYs). Hence, the burden of FBD (33 million DALYs) is of a similar order of magnitude as each of the 'big three' infectious diseases (HIV/AIDS, malaria and tuberculosis) and air pollution, but clearly lower than the burden of dietary risk factors or unimproved water and sanitation.

Our estimate of 29,000 deaths due to foodborne transmission of invasive non-typhoidal *S*. *enterica* only included infections in non-HIV infected individuals. Ao et al. [40] estimated there were approximately 680,000 deaths due to invasive non-typhoidal salmonellosis in 2010. Of these, approximately 350,000 would be due to foodborne transmission, assuming 52% of all non-typhoidal salmonellosis cases is transmitted by food [14]. Even though this high number of deaths among HIV infected people is not included in the FERG estimates of the burden of FBD, they would be preventable by food safety interventions.

### Limitations

Our study is subject to several limitations, notably due to uncertainties in the data limitations on burden estimates and attribution estimates. For most hazards (25 of the 31 studied), the 95% DALY uncertainty interval (UI) ranged from one-fourth to four times the median. The uncertainty was markedly greater for *E*. *multilocularis* (because of uncertainty in the attribution estimates), *E*. *granulosus* and *L*. *monocytogenes* (because of uncertainties in the imputation results). In low-income countries, where the burden is highest, data availability was generally most problematic. Furthermore, in these countries, the proportions of diseases transmitted by food, water and the environment are difficult to disentangle, as contaminated water may also result in contamination of foods. Due to these limitations, we were not able to present reliable estimates at country level, and elected to present results at subregion level.

For some hazards (e.g., *M*. *bovis* and *E*. *multilocularis*, aflatoxin and dioxins), incident illness is related to past exposures due to long incubation times of disease. For such hazards, the estimated burden reflects exposure dating back to the average incubation period of the disease rather than current exposure. For some hazards (e.g., dioxins), the impact on the child depends on the lifelong exposure of the mother.

Our estimates of the FBD burden are probably conservative, i.e., underestimates rather than overestimates. Limited resources and data obliged us to focus on only a subset of more than 100 hazards of potential relevance [41]. In particular, we did not include burden estimates for several chemicals (arsenic, cadmium, lead and methylmercury), because methods for estimation of the fraction of illnesses attributed to foodborne exposure to these chemicals are not readily available. Even for the hazards we have studied, it was not always possible to include all relevant disease outcomes in our estimates of burden. For example, we did not include functional bowel disorders as potential outcomes for enteric infections [42]. Inclusion of these outcomes would likely considerably increase the burden of enteric infections [43]. Aflatoxin burden was estimated using a counterfactual approach, estimating population attributable fractions from exposure assessment estimates and cancer potency factors, and applying these to WHO estimates for incidence and mortality by hepatocellular carcinoma. Risk assessment, as used to assess the burden of dioxins [19], has been proposed as an alternative basis for estimating this particular burden, and would result in considerably higher estimates of the burden of aflatoxin [44].

A further limitation of this study is that DALYs do not quantify the full societal impact of FBD. The economic burden (cost-of-illness, losses in the agricultural and food sectors and trade impacts) is also an important factor to consider in national and international decision-making. Also, the process of food production can cause human diseases by mechanisms other than direct transmission of pathogens through food. For example, animal husbandry is an important source of zoonotic disease agents that spread from pigs, poultry, cattle, etc, by direct contact or through the environment, and may also affect livestock health. It is increasingly necessary to consider holistically all aspects of food-related disease in a One Health Framework [45].

### Enforcing Food Safety Standards

Despite its data gaps and assumptions, this study presents the first ever estimates of the global burden of FBD and should serve as an important resource to focus activities that will reduce this burden. A sustainable, multi-sectoral response is needed from governments and international organizations to reduce the visible and ‘hidden’ burden; this includes enforcement of food safety standards and effective surveillance networks at country, regional and global levels. This will require a concerted effort by all stakeholders in the food chain, from primary production to consumers. The diversity of foodborne hazards suggests the need for a multi-faceted strategy, with priorities tailored to each region. While national studies may further refine these priorities and are highly recommended, the current findings could already be a basis for developing strategies at the global, regional and national levels.

The diversity of foodborne hazards and regional differences in their importance suggest the need for consideration of these estimates at the national or even subnational level. As one of its aims, the FERG has fostered national studies of the burden of FBD, and pilot studies have been conducted in Albania, Japan, Thailand and Uganda. The tools and protocols developed by the FERG to support such national studies emphasize the collation of local data to validate its regional estimates, the consideration of local hazards that may not have been addressed at a global level, and the translation of burden estimates into food safety policy. The estimates developed by this WHO initiative will be invaluable for countries where local data gaps prevent the development of a full picture of FBD.

The considerable difference in the burden of foodborne disease between low- and high-income regions suggests that a major proportion of the current burden is avoidable. The WHO is working with governments and partners, including food producers, caterers and consumers, to reduce food contamination throughout the food chain, and particularly at the point of consumption, to levels at which the exposure to pathogens and contaminants does not pose significant risks for human health. There is, therefore, an urgent need to develop cost-effective food hygiene interventions that can be implemented in resource-poor settings. This research and development should be informed by estimates of the burden of specific food vehicles, taking all hazards into account.

General principles for strengthening food safety systems have been suggested by the WHO; they include integrating food safety into nutrition and food security policies and programs, and fostering closer collaboration between the various sectors involved (agriculture, human health, animal health, trade, tourism, etc.). The WHO recommends governments put in place risk-based food control systems and implement international food safety standards as established by the Codex Alimentarius Commission [12]. Food handlers and consumers should handle and prepare food safely, practicing the WHO's 'Five Keys to Safer Food' and grow fruits and vegetables using the WHO's 'Five Keys to Growing Safer Fruits and Vegetables' to decrease microbial contamination [46].

FBDs are closely linked to poverty in developing countries but they are also a global public health issue because growing international trade increases the risk of contamination in transported foods; also, migration and travel can expose populations to new hazards. Achievement of the internationally agreed Millennium Development Goals and the proposed Sustainable Development Goals, including the overarching goals of poverty reduction, achieving food security and ensuring healthy lives, will depend in part on successful reduction of the burden of FBD.

## Supporting Information

S1 Table World Health Organization Foodborne Disease Burden Epidemiology Reference Group. Estimates of the global burden of foodborne disease, 2010.
(XLSX)

## Abbreviations

DALYs: Disability Adjusted Life Years
EPEC: Enteropathogenic *Escherichia coli*
ETEC: Enterotoxigenic *Escherichia coli*
FBD: Foodborne disease
FERG: Foodborne Disease Burden Epidemiology Reference Group
FOS: Department of Food Safety and Zoonoses (WHO)
GBD: Global Burden of Disease
SMPH: Summary measure of population health
STEC: Shiga-toxin producing *E*. *coli*
YLD: Years Lived with Disability
YLL: Years of Life Lost
